# Report of the procedure of voluntary interruption of pregnancy at a university hospital in Uruguay

**DOI:** 10.1590/S1518-8787.2016050006001

**Published:** 2016-06-28

**Authors:** Ana Bentancor, Ana Laura Hernández, Yamile Godoy, Juan J Dapueto

**Affiliations:** Departamento de Psicología Médica. Hospital de Clínicas. Facultad de Medicina. Universidad de la República. Montevideo, Uruguay

**Keywords:** Abortion, Induced, legislation & jurisprudence, Abortion, Legal, Patient Care Team, Program Evaluation, Health Human Resource Training, Comprehensive Health Care

## Abstract

**OBJECTIVE:**

To describe the constitution and operation of a voluntary interruption of pregnancy team of a university hospital, from the outlook of the mental health team.

**METHODS:**

In this case study, the following aspects were analyzed: 1) historical background; 2) implementation of Law 18,897 of October 22, 2012; and 3) functioning of the program at the Hospital de Clínicas of the Facultad de Medicina (Universidad de la República, Uruguay), taking into account three dimensions: structure, process, and results.

**RESULTS:**

Between December 2012 and November 2013, a total of 6,676 voluntary interruptions of pregnancy were reported in Uruguay; out of these, 80 were conducted at the Hospital de Clínicas. The patients’ demographic data agreed with those reported at the national level: Of the total patients, 81.0% were aged over 19 years; 6.2% decided to continue with the pregnancy; and only 70.0% attended the subsequent control and received advice on contraception.

**CONCLUSIONS:**

In its implementation year in Uruguay, we can assess the experience as positive from the point of view of women’s health. Our experience as a mental health team at the Hospital de Clínicas, inserted into the multidisciplinary voluntary interruption of pregnancy team, is in the process of assessment and reformulation of practices.

## RESUMEN

**OBJETIVO:**

Describir la conformación y funcionamiento de un equipo de interrupción voluntaria del embarazo de un hospital universitario, desde la mirada del equipo de salud mental.

**MÉTODOS:**

En este estudio de caso, se analizan los siguientes aspectos: 1) antecedentes históricos; 2) implementación de la Ley 18.897 de 22 de octubre de 2012; y 3) funcionamiento del programa en el Hospital de Clínicas de la Facultad de Medicina (Universidad de la República, Uruguay), teniendo en cuenta tres dimensiones (estructura, proceso y resultados).

**RESULTADOS:**

Entre diciembre de 2012 y noviembre de 2013, se reportaron en Uruguay un total de 6.676 interrupciones voluntarias del embarazo; de ellas, 80 se llevaron a cabo en el Hospital de Clínicas. Los datos sociodemográficos de las pacientes concordaron con los reportados a nivel nacional: 81,0% de las pacientes que consultaron era mayores de 19 años; 6,2% decidieron continuar con el embarazo; solo 70,0% concurrieron al control posterior y recibieron asesoramiento sobre anticoncepción.

**CONCLUSIONES:**

Al año de su implementación, en Uruguay, podemos evaluar la experiencia como positiva desde el punto de vista de la salud de las mujeres. Nuestra experiencia como equipo de salud mental en el Hospital de Clínicas, inserto dentro del equipo multidisciplinario de interrupción voluntaria del embarazo, se encuentra en pleno proceso de evaluación y reformulación de prácticas.

## INTRODUCTION

In ancient societies, abortion did not mean a moral problem. Since the early days of Christianity, the ethical and moral aspects of abortion began to be discussed. In the year of 1869, Pope Pius IX proclaimed the theory of immediate hominization or ensoulment (the moment at which a human being gains a soul) at the moment of conception, an idea that has been maintained by the Catholic Church to the present day^6^. Since the early 20^th^ century, in Uruguay, abortion was criminalized, except in four situations: rape, honor of the woman’s husband or father, economic distress, and serious risk to the woman’s health.

Being considered a crime, most of the consultations had to do with post-abortion complications, and was followed by a complaint to the health authority. The fear of penalty led to the late appointment and high morbidity and mortality rates^1^.

In 2004, ISCAPCR (Health Initiatives Against Unsafe Abortion) model^2^ was implemented, which proposed two interviews: a pre-abortion, to control pregnancy and inform and prevent the risk of the procedure; and a post-abortion, to rehabilitate the patient holistically and implement measures of contraception. This process culminated with the approval of Law 18,987, which enables the voluntary interruption of pregnancy (VIP) within the first 12 weeks of gestation for the Uruguayan women, minors or adults, and for foreign women residing in the country. The term extends to 14 weeks in case of rape, and throughout the pregnancy, in case of risk to the woman’s health or fetal abnormalities incompatible with life outside the womb.

VIP healthcare teams have a gynecologist, a specialist in mental health, and a specialist in the social area. The procedure consists of four stages^1^.

- VIP 1: appointment with physician or midwife, for information and performance of an ultrasound scan and blood group.
- VIP 2: interviews with the multidisciplinary team for information and advice for making a final decision. Five days of reflection must elapse before the VIP 3.
- VIP 3: if the patient confirms her decision, the gynecologist prescribes the medication (mifepristone and misoprostol). The procedure is usually performed on an outpatient basis, but it may be on an inpatient basis. Otherwise, a perinatal record is made. This is the only stage in which the gynecologist can have a conscientious objection.
- VIP 4: appointment with gynecologist for control and diagnosis of possible complications and to provide advice on contraception. If the woman fails to attend her appointment, she is contacted by telephone.

The purpose of this study was to describe the operation of the VIP healthcare team of the University Hospital, from the perspective of mental health professionals. The aim of this communication is to provide local and regional working groups with relevant information about this first stage of work, since Uruguay is the first country in the region to implement a program of sexual and reproductive health of this kind.

## METHODS

In this case study, we conducted the analysis of the structure, process and results of the VIP program of the Hospital de Clínicas of the Facultad de Medicina, Universidad de la República (UdelaR), in Uruguay, after its first year of application^3^.

Patients were assisted in the Gynecological Polyclinic, according to the four stages provided for in the regulation.

In the psychological interview, the aim was to facilitate the decision-making process autonomously, avoiding giving advice and value judgments as well as to determine any support resource requirements. A guideline for the performance of the mental health professional (Table 1) was created.

**Table 1:** Guideline for the performance of the mental health professional of the VIP advice teams.

Specific objective	Provide elements that promote decision making ability autonomously, and identify any specific support resource requirements
Joint objectives of the interdisciplinary team	Detect biopsychosocial risk situations and manage appropriate actions Assess the woman’s desire to call the father Enable possible new appointment before the fifth day, if desired
Guideline for the interview	
General climate of the interview	• Avoid delayed care • Promote a space with adequate privacy
	• Ensure a climate of acceptance
	• Ensure confidentiality
	• Suggest and give the option of being accompanied by a supportive person during the process
	• Always use the term voluntary interruption of pregnancy
	• Avoid using terms with moral, religious, or philosophical connotations
	• Do not be mobilizer (interview style)
	• Avoid confrontations that promote distress
Topics to discuss	• Talk about the decision-making process
	• Assess mental health status
	• Assess personal history related to mental health
	• Assess the relationship with the father
Interventions	• Contribute to psychosocial diagnosis •
	Articulate in the risk profile and manage actions
	• Inform that emotional pain is part of a normal process
	• Connect with mental health team, if necessary
	• Make a new appointment if the patient wants
Neutrality and excluded topics	• Do not offer advice
	• Do not alarm
	• Do not impose philosophical or personal convictions
	• Refrain from value judgments
	• Refrain from preventing or promoting the VIP
	• Do not perform a psychotherapeutic interview

VIP: voluntary interruption of pregnancy

All information was gathered from medical records following the registration form provided by the Ministry of Public Health, which includes an informed consent form. In the interview by the mental health professional, the following issues were taken into account: personal psychiatric history, stressful life events in the past year, social and family support, and the presence of psychosocial problems.

## RESULTS

Between November 2012 and November 2013, 80 patients attended the VIP Polyclinic of the Hospital de Clínicas. Sociodemographic characteristics are listed in Table 2. Average age was 27 years, and most of the women were single. Out of the patients with a psychiatric history (n = 8, 10.0%), most were associated with depression.

**Table 2:** Sociodemographic and gyneco-obstetric data. Hospital de Clínicas, Montevideo, Uruguay, 2012-2013.

Age	Average: 27 years old Minimum 16 years old Maximum 42 years of old	SD: 7.3
	
	n	%
Occupation		
Housework	22	31.9
Employee	13	18.8
Student	12	17.4
Unemployed person	8	11.6
No data	14	20.3
Marital status		
Married or common-law union	16	23.2
Single	40	58.0
Divorced or separated	4	5.8
No data	9	13.0
Education level		
Primary	1	1.4
Secondary	7	10.1
Tertiary	4	5.8
No data	57	82.6
Origin		
Montevideo	54	78.3
Inland	6	8.7
No data	9	13.0
Accompanied by		
Nobody	23	33.3
Couple	10	14.5
Children	2	2.9
Father or mother	3	4.3
Other	4	5.8
No data	27	39.2
Psychiatric history		
Depression	6	8.7
Addictions	1	1.4
Domestic violence	3	4.4
SA	2	2.9
Others	1	1.4
No history	5	7.3
No data	51	73.9
Reason for VIP		
Unwanted pregnancy	36	52.2
Chronic pathology	6	8.7
Family difficulties	1	1.4
Others	1	1.4
No data	20	36.3
Date of gestation (week)		
< 5	1	1.5
5	6	8.7
6	9	13.0
7	14	20.3
8	9	13.0
9	9	13.0
10	8	11.6
11	2	2.9
12	2	2.9
13	3	4.4
No data	6	8.7
Contraception		
Yes	24	34.8
No	3	4.3
No data	42	60.8
Contraception method		
Condom	9	37.5
OC	10	41.6
IUD	5	20.8

SD: standard deviation; SA: suicide attempts; VIP: voluntary interruption of pregnancy; OC: oral contraceptives; IUD: intrauterine device

Out of the 80 patients: three (3.8%) were not pregnant; five (6.3%) continued with the pregnancy; four (5.0%) exceeded the 12 weeks of gestation at the time of the first appointment; 11 (13.8%) were lost in the follow-up; and 57 (71.3%) attended the VIP 2 appointment.

Gynecologic and obstetric data are listed in Table 2. The most common reason for interruption was unwanted pregnancy, followed by: chronic diseases that can affect the health of the mother and child; stage of life with plans incompatible with motherhood; and family difficulties.

Out of the 57 patients who attended the VIP 2 appointment, 50 (87.7%) completed the VIP 3 appointment and, therefore, received the medication. Out of them, 41 (82.0%) continued the process on an outpatient basis, and 9 (18.0%) were admitted to the hospital. In three cases (6.0%) the procedure had to be completed with curettage.

Thirty-seven patients (74.0%) attended the VIP 4 appointment and 13 (26.0%) were lost in the follow-up. Mortality was zero for the study group.

## DISCUSSION

After a year of the implementation of the VIP law, a total of 6,676 procedures (41.0% in the public subsector and 59.0% in the private subsector^7^) were reported. From the cases, 64.0% corresponded to the capital Montevideo and 36.0% to inland. The data gathered are in compliance with those reported at the national level, with: 82.0% of procedures in women over 20; 6.2% of cases continuing with the pregnancy; and only 70.0% attending VIP 4^7^. We observed that unwanted pregnancies often resulted from failure or lack of contraception. It has been shown in previous studies that 43.0% of unplanned pregnancies are the result of improper use of contraceptive methods, and that 52.0% are caused by lack of use^7^.

The constitution of multidisciplinary teams was different in the different institutions involved. In many cases, they did not includ gynecologists, who claimed conscientious objections (up to 30.0% of them in some departments in the country). As it has happened in other countries, this principle has been difficult to determine, so that it did not incur in non-compliance with the principle of autonomy^4^.

In the Hospital de Clínicas of UdelaR, a work team with high technical and human quality, seeking a care based on respect, containment, and adequate and necessary information has been consolidated. The integration with the VIP team helped us to better understand women exposed to this problem. Although the psychological interview does not arise from the demand of patients, it is received with willingness and valued as an opportunity. In this sense, we consider important to continue opening lines of work that allow us to explore some aspects related to women’s mental health in specific relation to the VIP procedure. Some studies show that women who performed an abortion have similar or lower levels of depression and anxiety than women who were denied the abortion by advanced gestational age^5,8^. These results then do not support the idea that abortion is one of the causes of main incidence of mental health problems. It would be very useful, to bring us closer to this perspective regarding population welfare, to be able to implement the most appropriate intervention strategies.

An additional aspect to consider is the emotional impact on teams. The following has been useful in this regard: rotation of tasks; voluntary participation in the VIP team; existence of a space for exchange and supervision; and academic support of the institution.

We have also identified areas to improve program implementation. No additional human resources were made available, so an extra effort by professionals was required. The facility available for care of patients is shared with the gynecologic and obstetric polyclinic, where sometimes interviews are interrupted. As part of this limitation, the registration was affected in the first stages, which explains the lack of data on the analyzed forms. From the point of view of the formation of human resources, we consider the low presence of stages of interinstitutional exchange and discussion as a difficulty.

Our experience as specialists in mental health within an academic and multidisciplinary VIP team, is in process of assessment, reformulation and adaptation of practices.

The work presented here has several limitations. This is a case study, merely descriptive of the characteristics of the patients treated in the mental health facility; in addition, it provides summarized information on the institutional procedure. However, its function is to communicate an innovative experience in our country.

In terms of results, it is preliminary data that require further exploration. We think that it is an innovative experience for a South American country, which can be input for professionals from countries of the region in which the theme begins to be discussed.
